# Effectiveness of a Pharmacogenetic Tool at Improving Treatment Efficacy in Major Depressive Disorder: A Meta-Analysis of Three Clinical Studies

**DOI:** 10.3390/pharmaceutics11090453

**Published:** 2019-09-02

**Authors:** Silvia Vilches, Miquel Tuson, Eduard Vieta, Enric Álvarez, Jordi Espadaler

**Affiliations:** 1 AB-Biotics, S.A., Av. de la Torre Blanca 57, 08172 Sant Cugat del Valles, Barcelona, Catalonia, Spain; 2 Department of Psychiatry and Psychology, Institute of Neuroscience, C/ Villaroel, 170, 08036 Barcelona, Catalonia, Spain; 3 Institut d’Investigacions Biomèdiques August Pi I Sunyer (IDIBAPS), University of Barcelona, C/ Rosselló, 149, 08036 Barcelona, Catalonia, Spain; 4 Centro de Investigación Biomédica en Red de Salud Mental (CIBERSAM), Av. Monforte de Lemos, 3-5, 28029 Madrid, Spain; 5 Servei de Psiquiatria, Hospital de la Santa Creu i Sant Pau, C/ Sant Antoni Maria Claret, 167, 08025 Barcelona, Catalonia, Spain; 6 Institut d’Investigacions Biomèdica Sant Pau, Universitat Autònoma de Barcelona, 08025 Barcelona, Catalonia, Spain

**Keywords:** antidepressants, depression, genetic, pharmacogenetics, psychiatry, randomized controlled trials

## Abstract

Several pharmacogenetic tests to support drug selection in psychiatric patients have recently become available. The current meta-analysis aimed to assess the clinical utility of a commercial pharmacogenetic-based tool for psychiatry (Neuropharmagen^®^) in the treatment management of depressive patients. Random-effects meta-analysis of clinical studies that had examined the effect of this tool on the improvement of depressive patients was performed. Effects were summarized as standardized differences between treatment groups. A total of 450 eligible subjects from three clinical studies were examined. The random effects model estimated a statistically significant effect size for the pharmacogenetic-guided prescription (*d* = 0.34, 95% CI = 0.11–0.56, *p*-value = 0.004), which corresponded to approximately a 1.8-fold increase in the odds of clinical response for pharmacogenetic-guided vs. unguided drug selection. After exclusion of patients with mild depression, the pooled estimated effect size increased to 0.42 (95% CI = 0.19–0.65, *p*-value = 0.004, *n* = 287), corresponding to an OR = 2.14 (95% CI = 1.40–3.27). These results support the clinical utility of this pharmacogenetic-based tool in the improvement of health outcomes in patients with depression, especially those with moderate–severe depression. Additional pragmatic RCTs are warranted to consolidate these findings in other patient populations.

## 1. Introduction

Major depression (MDD) is a highly prevalent disorder that is rapidly becoming a major public health problem. It was recently estimated that more than 300 million people worldwide suffer from depression—equivalent to 4.4% of the world’s population [1]. Globally, depression constitutes one of the leading causes of disability and causes high health care expenditures [2,3,4]. Despite the growing number of commercialized psychotropic drugs, psychiatry faces the challenge to improve the current “trial-and-error” model of treatment prescription. Response rates of individual antidepressants are low, especially in patients with mild depression [5,6]. Treatment-resistant depression (TRD)—usually defined as the failure of two successive pharmacological products at an adequate dosing and duration—represents at least 20–30% of MDD cases [7]. Of note, real-life effectiveness is further limited by low treatment adherence [8]. High rates of treatment failure and drug-induced adverse effects bring with them economic as well as social burden [7,9,10]. 

Genetic variation has been shown to contribute to drug response, metabolism and safety. In an effort to standardize and facilitate implementation of pharmacogenetics into clinical practice, international expert consortia—such as the Clinical Pharmacogenetics Implementation Consortium (CPIC)—are committed to the issuance and update of peer-reviewed, evidence-based clinical guidelines with gene/drug pairs, grading of evidence, standardized terminology and clinical recommendations [11,12,13,14,15,16,17]. Medicine agencies are also aware of the genetic impact in drug response and the number of approved drug labels that incorporate information on genetic biomarkers is consequently growing [18,19]. This has led to an increasing number of pharmacogenetic-based decision support tools and tests commercially available. However, associations between certain genetic variations and a clinical outcome (i.e., clinical validity) are necessary but not sufficient to support using a specific pharmacogenetic (PGx) tool, as indicated by the ACCE (Analytical validity, Clinical validity, Clinical usefulness, Ethical and legal implications) model [20]. For an adequate clinical implementation of pharmacogenetic testing, clinical utility must be demonstrated. In other words, interventional randomized controlled trials (RCTs) must be conducted to demonstrate that a particular PGx tool, defined by a set of genetic variations and an algorithm translating them into a defined set of clinical recommendations, results in improved health outcomes compared to the usual clinical practice. Moreover, it has been recommended that such trials should be conducted with a patient population representative of real practice, i.e., pragmatic trials [21]. This is supported by the observation of significant differences on intrinsic antidepressant efficacy between trials conducted under naturalistic conditions and those conducted with highly selected, phase-III-like patient populations [22]. 

Neuropharmagen^®^ (AB-Biotics SA, Barcelona, Spain) is a commercial PGx-based tool that uses a proprietary approach to generate integrated recommendations based on: (i) available published pharmacogenetic guidelines (e.g., CPIC guidelines for dose adjustments based on combined genotypes), PGx information on FDA-approved drug labeling and other selected clinical studies, and (ii) a safety-first approach to rank genetic variations contributing to higher risk of adverse effects above other variations. Two prospective, parallel-arm clinical studies and one retrospective study have assessed the clinical utility of this PGx-based tool in the pharmacological treatment of patients with MDD. These studies were a twelve-week, randomized, double-blind, multicentric clinical trial (AB-GEN study, *n* = 280) [23], an eight-week, randomized, single-blind clinical trial (Korean study, *n* = 100) [24], and a twelve-week follow-up, retrospective, naturalistic, multicentric trial (GENEPSI study, *n* = 70) [25]. The main variable used to evaluate efficacy varied across the three studies. Common to all three was the assessment of treatment efficacy by means of the clinician-rated Clinical Global Impression of Severity scale (CGI-S, [26]), to compare patients whose clinician used the pharmacogenetic tool to guide medication selection (PGx-guided) with patients that were treated following the usual clinical practice. The two prospective randomized clinical trials additionally evaluated patient improvement in depression symptoms using the 17-item Hamilton Depression Rating Scale (HDRS-17, [27]).

Here, we present a meta-analysis of the three clinical studies that have evaluated the clinical utility of the pharmacogenetic information provided by this PGx-based tool in the selection of pharmacological treatments in depressive patients as compared to the usual clinical practice.

## 2. Materials and Methods 

The Preferred Reporting Items for Systematic Reviews and Meta-analyses (PRISMA) recommendations specifically applying to meta-analyses were followed [28].

### 2.1. Data Collected

All subjects in the three studies were 18 years old and over, with a primary diagnosis of major depressive disorder, a CGI-S score ≥3 as rated by the clinician, and either required medication *de novo* or a substitution or addition of a drug. The mean CGI-S and HDRS-17 scores at baseline did not differ between PGx-guided and unguided groups in any of the three studies. Patients with concomitant psychiatric diagnosis were not excluded except for the Korean study. 

Data collection included the following items:

- Design and duration of each study;
- Patients’ characteristics as per treatment group: sample size, mean age, gender distribution, ethnicity, psychiatric comorbidities, baseline and final visit CGI-S scores, and baseline and final visit HDRS-17 scores when available.

All three clinical studies were approved by the Institutional Review Boards (IRB) of the corresponding participating centers and were conducted in compliance with the Declaration of Helsinki. The GENEPSI study protocol was approved by the IRB of Hospital Clínico San Carlos in Madrid, Spain (protocol code: ALM-PSI-2013-01, approval date: 20/12/2013); the AB-GEN study protocol was approved by the IRB of Hospital Clínic de Barcelona, Spain, acting as a centralized reference IRB, as well as the IRB of each participating hospital (protocol code: AB-GEN-2013, approval date: 15/11/2013); the Korean study protocol was approved by the Catholic University of Korea Bucheon St. Mary’s Hospital IRB (approval number: HC16EIMI0015, approval date: 02/03/2016). Written informed consent was obtained from all participants of the AB-GEN and the Korean studies before enrolment. In accordance with the standard policy of AB-Biotics, all patients of the GENEPSI study provided written informed consent for the genetic testing.

Following informed consent, all participants provided a saliva sample for DNA extraction and genotyping of selected genetic variants. Details on the laboratory analyses are described elsewhere [23]. 

The pharmacogenetic data derived from the analysis of genetic polymorphisms in 30 genes associated with drug efficacy, metabolism and specific adverse effects (Table S1). The Neuropharmagen^®^ report (AB-Biotics S.A., Barcelona, Spain) was accessible through a web-based computer-aided system, and provided information for a variety of antidepressants, antipsychotics, mood stabilizers and other central nervous system drugs (Figure S1). The PGx report was composed of: (1) a summary table based on a “safety first” approach that prioritizes alerts using a traffic light color code: red indicates an increased risk of adverse drug reactions, yellow highlights the need for drug dose monitoring and/or less likelihood of positive response, green is associated with an increased likelihood of positive response and/or lower risk of adverse drug reactions, and white indicates “use as directed”—no relevant genetic variants found, and (2) detailed PGx results and integrated drug-specific interpretations as per recommendations found in FDA-approved drug labeling [18] and pharmacogenetic guidelines [11,14,15], as well as information from selected clinical studies [29,30,31,32,33,34]. 

### 2.2. Outcomes

The measure of efficacy of the intervention (effect size) was computed as standardized differences, using Hedge’s improved estimate of Cohen’s *d* [35], from the clinician-rated CGI-S score change from baseline to the final visit of each study. The CGI-S was the only variable common to all three studies included. The two RCT also evaluated symptom remission with the HDRS-17. Therefore, a secondary endpoint of this meta-analysis was the effect size calculated from HDRS-17 score change from baseline to the final visit of each study.

### 2.3. Statistics

Continuous variables were summarized as mean ± standard deviation (SD). Categorical variables were expressed as number and percentage. Baseline characteristics of individual studies were compared using SPSS version 20 (IBM Corp., Chicago, IL, USA).

Random effects meta-analysis was conducted using the Meta [36] and Metafor [37] packages and the application R studio version 1.2.1335 [38] of *R* statistical software version 3.6.0 [39]. Standardized mean differences (SMD), and their respective 95% confidence intervals (CIs), were calculated from clinical response data (CGI-S and HDRS-17 score change) for each individual study as well as the combination of all. A positive effect size with a 95% CI excluding zero indicated that treatment in the PGx-guided arm was superior to the control group (a *p*-value < 0.050 was considered statistically significant). 

Statistical heterogeneity across studies was determined by the Inconsistency statistic (*I*^2^). *I*^2^ is the proportion of total variation attributed to differences between studies, and it is usually interpreted based on the values 25%, 50% and 75%, to identify a low, moderate and high heterogeneity, respectively [40]. As there was significant statistical heterogeneity, pooled effect sizes for each clinical endpoint were calculated using random effects models, and the significance of each pooled effect size was determined using Z-tests. 

Finally, odds-ratios (ORs) were estimated from standardized differences in the continuous variables analyzed (CGI-S and HDRS-17 score change) using the logistic approximation method developed by Hasselblad and Hedges [41,42].

### 2.4. Assessment of Bias

The overall risk of bias of the RCTs included was assessed by S.V. and J.E. based on the six recommended domains by the Cochrane risk of bias tool [43]: sequence generation, allocation concealment, blinding, incomplete outcome data, selective outcome reporting, and other sources of bias. The Risk Of Bias In Non-randomized Studies—of Interventions (ROBINS-I) tool [44] was used to evaluate risk of bias of the GENEPSI study. 

## 3. Results

The main characteristics of the three clinical studies evaluating the clinical outcomes of Neuropharmagen^®^-guided vs. unguided prescription in patients with MDD are summarized in Table 1. The studies included a total of 450 patients with MDD as the primary diagnosis, a median age of 50 years (range 28.1–70.4) and a female–male ratio of 2.6:1. The Korean and the GENEPSI studies required failure of at least one previous medication treatment because of lack of adequate efficacy and/or tolerability, whereas naïve patients were not excluded in the AB-GEN study. The average of previous failed antidepressant trials for the current episode was 2.6 ± 2.2 in the AB-GEN study and 2.3 ± 1.9 in the Korean study. The two RCTs allocated patients meeting the inclusion criteria to either PGx-guided or treatment as usual (TAU) groups using a computer-generated random list, that unlocked or locked the clinician’s access to the PGx results, respectively. The Korean study followed subjects for eight weeks after randomization, while the AB-GEN study followed the participants for twelve weeks. Variables for patient clinical assessment (indicated in Table 1) were recorded at randomization, mid of the study period, and final visits. The GENEPSI retrospective study [25] established the baseline visit as the one in which the saliva sample from the patient was collected and a follow-up visit 12 weeks after the baseline visit. In this study, patients were retrospectively classified into one of two groups: (1) patients whose psychiatrist prescribed the pharmacological treatment according to PGx-based recommendations; or (2) patients whose treatment did not follow the PGx recommendations—not including or removing drugs with green alerts, and/or including medications with yellow and/or red alerts—(see Figure S1 for an example of the PGx report). 

The primary variable differed among the three trials: CGI-S, PGI-I (Patient Global Impression of Improvement) and HDRS-17 were used by the GENEPSI, the AB-GEN and the Korean studies, respectively, but the CGI-S was assessed by all the studies to evaluate patients’ improvement. The two RCTs, including only patients with a primary diagnosis of MDD, also assessed the HDRS-17 as response variable. Conversely, the retrospective trial only assessed the CGI-S because the studied population included patients with different primary psychiatric diagnoses—MDD, bipolar disorder, schizophrenia and generalized anxiety disorder. For the purposes of this meta-analysis, only data from those patients with a primary diagnosis of MDD has been considered. The mean CGI-S and HDRS-17 scores at baseline did not differ between PGx-guided and unguided groups in any of the three studies (Table 2). Baseline severity as evaluated by the CGI-S was similar across the three trials. However, when depressive symptoms were evaluated with the HDRS-17, patients from the AB-GEN study showed an average score of 19.2 ± 5.8 whereas participants of the Korean study presented a mean HDRS-17 score of 23.8 ± 4.8 (*p*-value < 0.001). The AB-GEN study recruited patients within all the range of severities (including mild depression), while the Korean study required participants with moderate–severe depression at baseline as per HDRS-17. 

The results of the risk of bias assessment for the AB-GEN and Korean RCTs, and the non-randomized GENEPSI study are presented in Table 3 and Table 4, respectively. The AB-GEN and Korean RCTs had a similar risk of bias. Selection, attrition and reporting biases were considered low. However, since treating clinicians in both RCTs were unblinded, there was a potential for performance and detection biases. The Korean study was not sponsored by industry, but the AB-GEN trial involved funding by the company in charge of developing the PGx tool tested. Regarding the quality of the GENEPSI study, its retrospective design allowed to reduce the risk of bias of typical open-label studies. Patients were balanced regarding baseline severity (CGI-S), age, sex, substance abuse and concomitant diseases. The intervention as well as baseline and follow-up visits were clearly defined. All prespecified outcomes were reported and patients lost to follow-up were evenly distributed.

**Figure 1:**
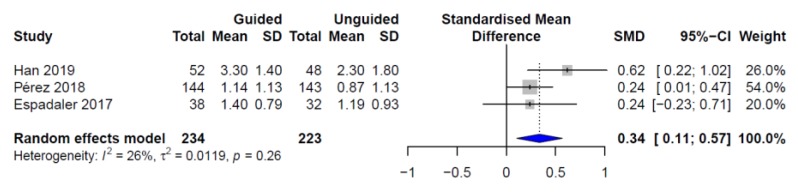
Individual and pooled effect sizes for clinical response based on CGI-S score change.

**Figure 2:**
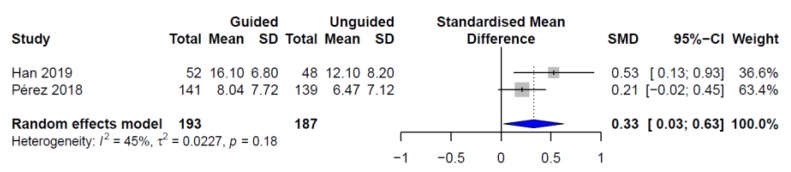
Individual and pooled effect sizes for clinical response based on HDRS-17 score change.

Based on the results of the three studies (*n* = 450), the random effects model estimated an statistically significant effect size—as computed from the clinician-rated CGI-S score change—of *d* = 0.34 (95% CI = 0.11–0.56, *p*-value = 0.004) for the PGx-guided prescription (Figure 1), with a heterogeneity of *I*^2^ = 23.13%. Based on the results of the two RCTs (i.e., AB-GEN and Korean studies, *n* = 380), the random effects pooled standardized mean difference (SMD) computed from the HDRS-17 score change also significantly favored the PGx-guided drug selection (*d* = 0.33, 95% CI = 0.03–0.63, *p*-value = 0.030) (Figure 2). Moderate heterogeneity was observed in this analysis (*I*^2^ = 44.8%). In order to address whether the estimated effect size varied in relation to depression severity, a sub-analysis was performed including the Korean population together with those participants from the AB-GEN study with a baseline HDRS-17 score >17 (i.e., patients with moderate–severe depression, *n* = 287). When patients with mild depression were excluded from the combined analysis based on the HDRS-17 score change, the pooled estimated effect size increased to 0.42 (95% CI = 0.19–0.65, *p*-value = 0.004). 

The estimated odds (OR) of clinical response for the PGx-guided vs. unguided prescription— calculated from the SMD computed from the CGI-S score change—was 1.84 (95% CI = 1.27–2.81). Similarly, an OR = 1.81 (95% CI = 1.05–3.12) was estimated from the SMD computed from the HDRS-17 score change, which increased to 2.14 (95% CI = 1.40–3.27) when only patients with moderate–severe depression (as per a HDRS-17 score > 17) were considered.

## 4. Discussion

To date, three studies on the clinical utility of PGx-guided prescription with Neuropharmagen^®^ in adult patients with MDD have been published [23,24,25]. The combined analysis of the results from these three studies demonstrates that PGx-guided treatment is associated with a higher effectiveness in terms of patients’ symptoms improvement, as assessed by the mean CGI-S and HDRS-17 score changes, compared to the usual clinical practice. Pooled effect sizes computed from the two scales were similar (i.e., *d* = 0.34 from the CGI-S score change and 0.33 from the HDRS-17 score change), supporting the robustness of the results. These effect sizes correspond to approximately a 1.8-fold increase in the odds of clinical response for PGx-guided vs. unguided drug selection. A similar overall effect size for antidepressants, as examined in acute-phase III trials, has been estimated (*d* = 0.30) [45,46]. It is noteworthy that while antidepressant phase III trials are placebo controlled, the trials included in the current combined analysis were controlled with a population of subjects being actively treated with antidepressants and other psychoactive drugs, without restriction in type, number and dose of said drugs. 

RCTs in patients with MDD often exclude subjects with clinical characteristics common to many patients seen in usual clinical settings. Several studies indicate that 65–90% of patients presenting for treatment of depression would be excluded from a RCT [47,48,49]. Comorbid psychiatric conditions are among the most prominent reasons for excluding patients. Paradoxically, about 60–70% of depressed patients have at least one concurrent psychiatric disorder, while 30–40% have two or more, being anxiety disorders and substance use disorders the most commonly observed [50,51,52,53,54]. Additionally, although it has been generally suggested that a score of ≤7 in the HDRS-17 is indicative of depression remission [55], a score over 17–19 is usually required for inclusion in a RCT since it has been observed that patients with higher disease severity at baseline respond better to treatment [6,56]. Overall, this practice of enrolling highly selected patients has been shown to significantly overestimate the benefit derived from antidepressant treatment [5]. Given that evidence of antidepressant efficacy has mainly been demonstrated in patients with moderate–severe depression, RCTs on the clinical impact of different pharmacogenetic tests in depressive patients have also established a HDS-17 ≥ 18 for patients inclusion in the study or the analysis of efficacy [57,58]. 

Among the three studies included in this meta-analysis, the AB-GEN trial, contributing with the highest number of patients (*n* = 280), was performed in a real-world clinical setting (Table 1). At randomization, the mean HDRS-17 score was 19.2 (± 5.8), and up to 17.8% of patients displayed depression of mild severity with a HDRS-17 score under 14, which was an exclusion criterium in the STAR*D study [59]. The average of previous failed antidepressant trials was 2.6 ± 2.2, ranging from 0–15. In addition, psychiatric comorbidities were not excluded (35.8% of patients presented with anxiety and 12.6% with substance use disorders) [23]. A recent reanalysis of the AB-GEN data showed that clinical utility of PGx was influenced by patient characteristics, including baseline severity [60]. In order to compare the results of the current meta-analysis with results from phase III clinical trials of antidepressant efficacy and phase III clinical trials of PGx-based prescription efficacy, that typically use more stringent inclusion/exclusion criteria, a sub-analysis was conducted including the Korean population together with those subjects from the AB-GEN study with a HDRS-17 score ≥18, yielding an increase of the SMD from 0.33 in the overall study population (including patients with mild depression) to 0.42 in the subpopulation with moderate–severe depression. The corresponding estimated OR for PGx-guided treatment was increased from 1.81 to 2.14 (95% CI = 1.40–3.27).

Other systematic reviews and meta-analyses combining results from clinical trials with different combinatorial PGx tests for psychiatry have suggested superiority compared with treatment as usual in adults with MDD [61,62]. However, different PGx tools are based on particular proprietary algorithms and differ in the set of genes and variants selected to produce the interpretation of the patient’s genetic profile, the way these are combined and translated to clinical recommendations, as well as in the form they display the results, among others. Therefore, any given PGx tool needs to be supported by specific RCTs [63,64]. Results of the present meta-analysis are in line with these previous analyses, but specifically demonstrate the clinical utility of pharmacogenetic testing with a specific tool over treatment as usual, especially in patients with moderate–severe depression. It is noteworthy that the clinical utility of this PGx tool is demonstrated in a sample population with a high prevalence of TRD. All participants in the Korean study had at least two failed antidepressant treatments for the current MDD episode, up to 65% of participants in the AB-GEN study were refractory at baseline, and the GENEPSI study required at least one previous failed treatment for patient inclusion. 

Some limitations that may affect the interpretations drawn from this meta-analysis have been identified. First, the small number of trials included, as well as the inclusion of an open-label retrospective study, may have an impact on the estimated pooled effect sizes. In order to mitigate this limitation, a sub-analysis including only data from the two RCTs was performed, yielding similar pooled effects. Second, the included clinical trials had potential sources of bias. Regarding quality assessment of the RCTs, selection bias was judged low, since both studies used a randomization list that stratified patients by study center in a 1:1 ratio to PGx-guided and control arms with the use of a computer-generated random list. Patients lost to follow-up were evenly distributed across the two arms of both RCTs, suggesting a low risk of attrition bias. Both RCTs reported all prespecified outcomes, indicating a low risk of reporting bias. Conversely, blinding can be considered a shortcoming of these trials. Although all participants included in the RCTs were blinded, the treating clinicians, who selected the drug treatment for patients in both groups, could not be blinded, introducing the potential risk of differences in attention. The AB-GEN study minimized this risk by introducing phone interviews by independent blinded raters, who collected the main variable (PGI-I). However, evaluation of longer scales, such as the HDRS-17, through phone interviews was considered inoperative. Face-to-face interviews by an independent rater would have allowed blinded evaluation of all secondary variables, but it would have also led to complicated logistics, probably increasing the number of dropouts. Other alternatives, such as using false genotyping data in the control group, have been suggested by some authors [65]. However, the risk of using a false genetic profile to select drugs makes this option ethically unacceptable. Regarding the quality of the open-label study, the overall risk of bias was judged low. When participants know they are being treated according to PGx-recommendations, there is an increased placebo effect resulting from the expectancy of being prescribed the right personalized treatment. In order to avoid this limitation, all participants included in the GENEPSI study had been retrospectively genotyped. There was a balanced distribution across both study arms regarding baseline severity (CGI-S), age, sex, substance abuse and concomitant diseases. The retrospective design of this study also allowed to minimize other risks of bias. The main variable (CGI-S) had been recorded in the clinical history by the treating clinician but prior to the protocol definition of the study. Although determined retrospectively, the intervention status was well defined and both study arms were balanced regarding patients’ characteristics (baseline severity as per CGI-S score, age, sex, substance abuse and concomitant diseases) and patients’ loss to follow-up. Baseline and follow-up visits were also clearly established, and all prespecified outcomes were reported. In terms of funding, two of the studies included (AB-GEN and GENEPSI studies) were partially funded by AB-Biotics (the company that developed and commercializes the PGx tool analyzed). In addition, personnel of AB-Biotics collaborated in the analysis of the results and preparation of the corresponding manuscripts. However, to our knowledge, the Korean study can be considered the first non-industry sponsored trial of a pharmacogenetic tool for psychiatry. Regarding ethnicity, most participants of the GENEPSI and the AB-GEN studies were of Caucasian origin. However, the 100 patients recruited by the Korean study (approximately 20% of the analyzed sample) were of Asian descent, thus providing proof of generalizability of the results to a different ethnicity than Caucasian. A final limitation identified was the possibility of having patients randomized to the PGx-guided group but being prescribed a drug treatment not in accordance with the recommendations provided by the PGx report. However, the case report form of the AB-GEN study allowed to control for this limitation, which represented 6.1% of the study group. 

## 5. Conclusions

The current meta-analysis demonstrates the clinical utility of a specific PGx-based tool in the guidance of drug selection in adult patients with moderate–severe MDD. However, due to the low number of studies included the generalizability of the present results may be limited. In order to support routine PGx testing in MDD patients, further pragmatic RCTs would be required to assess the actual impact of PGx testing in specific populations (e.g., patients with milder forms of depression, different ethnicities and/or age groups, among others). Given that a high percentage of patients that do not qualify for inclusion in an RCT still receive antidepressant treatment, inclusion/exclusion criteria in future trials should be reconsidered in order to bridge the gap between research and real clinical populations.

## Figures and Tables

**Table 1:** Characteristics of the patients and studies included.

Study	Study Design	Country	Sample Size	Demographics	Patient Characteristics
Korean study [24]	Eight-week, multicenter, prospective double-blind RCT Two arms: PGx-guided vs. unguided (TAU) Variables for clinical assessment: clinician-rated CGI-S, HDRS-17, FIBSER, PHQ-9/15, GAD-7, SDI	South Korea	100 (PGx-guided *n* = 52, TAU *n* = 48)	PGx-Guided vs. TAU: Sex (% females): 76.9 vs. 72.9 (ns) Age (years), mean (SD): 44.2 (16.1) vs. 43.9 (13.8) (ns) Ethnicity (%): Korean 100 vs. 100	Age ≥20 years Primary diagnosis of MDD by DSM-V CGI-S ≥3 despite current AD treatment at proper dosage and duration (at least 6 weeks) or intolerance Moderate–severe depression at baseline as per HDRS-17 Previous failed antidepressant trials, mean (SD): 2.3 ± 1.9 Patients with substance abuse or hospitalized within 8 weeks prior to study entry were excluded
AB-GEN study [23]	Twelve-week, multicenter, prospective double-blind RCT Two arms: PGx-guided vs. unguided (TAU) Variables for clinical assessment: PGI-I, clinician-rated and patient-rated CGI-S, HDRS-17, FIBSER, SDI, SATMED-Q	Spain	280 (PGx-guided *n* = 136, TAU *n* = 144)	PGx-Guided vs. TAU: Sex (% females): 63.9 vs. 63.4 (ns) Age (years), mean (SD): 51.74 (12.02) vs. 50.74 (13.12) (ns) Ethnicity (%): Caucasian 93.5 vs. 91.3, Latin American 4.5 vs. 6.2, Other 2.0 vs. 2.5 (ns)	Age ≥18 years Primary diagnosis of MDD by DSM-IV CGI-S ≥4 Requiring medication de novo or a substitution or addition of an AD Previous failed antidepressant trials, mean (SD): 2.6 ± 2.2 18% of patients had borderline depression at baseline (HDRS score <14) 13% of patients with known substance abuse at baseline No patients hospitalized at baseline
GENEPSI study [25]	Twelve -week, multicenter, retrospective, observational, naturalistic study Two groups: treatment following vs. not following PGx-recommendations Variables for clinical assessment: clinician-rated CGI-S, data on adverse effects extracted from medical records	Spain	70 (PGx-guided *n* = 38, unguided *n* = 32)	PGx-Guided vs. unguided: Sex (% females): 76.3 vs. 81.25 (ns) Age (years), mean (SD): 54.3 (14.5) vs. 55.2 (15.2) (ns) Ethnicity: mainly Caucasian	Age ≥18 years Psychiatric diagnosis according to ICD-10 Failure of the previous treatment (lack of efficacy and/or poor tolerability) CGI-S ≥3 30% of patients hospitalized at baseline 10% of patients with known illicit substance abuse HDRS-17 not assessed

AD: Antidepressant Drug; CBT: Cognitive-Behavioral Therapy; CGI-I: Clinical Global Impression of Improvement; CGI-S: Clinical Global Impression of Severity; DSM: Diagnostic and Statistical Manual of Mental Disorders; ECT: Electroconvulsive Therapy; FIBSER: Frequency, Intensity and Burden of Side Effects Ratings; GAD-7: General Anxiety Disorder-7; HDRS-17: 17-Item Hamilton Depression Rating Scale; ICD: International Statistical Classification of Diseases and Related Health Problems, 10th Revision; MDD: Major Depressive Disorder; ns: Not Statistically Significant (*p*-value >0.05); PGI:-I: Patient Global Impression of Improvement Scale; PGx: Pharmacogenetics; PHQ-9/15: 9-Item/15-Item Patient Health Questionnaire; RCT: Randomized Controlled Trial; SATMED-Q: Treatment Satisfaction with Medicines Questionnaire; SD: Standard Deviation; SDI: Sheehan Disability Inventory; TAU: Treatment as Usual.

**Table 2:** Baseline CGI-S and HDRS-17 scores in the pharmacogenetic-guided and control groups in the three clinical studies.

Study	Variable	PGx-Guided	Control	*p*-Value
Korean study [24]	CGI-S, mean ± SD	4.90 ± 0.80	4.60 ± 0.70	0.063
	HDRS-17, mean ± SD	24.50 ± 4.60	23.10 ± 5.00	0.159
AB-GEN study [23]	CGI-S, mean ± SD	4.50 ± 0.62	4.40 ± 0.57	0.166
	HDRS-17, mean ± SD	19.47 ± 5.96	19.01 ± 5.71	0.482
GENEPSI study [25]	CGI-S, mean ± SD	4.29 ± 0.57	4.26 ± 0.72	0.836
	HDRS-17, mean ± SD	na	na	na

na: not assessed.

**Table 3:** Assessment of the risk of bias of the Korean and AB-GEN randomized controlled trials.

Bias	Korea Study [24]	AB-GEN Study [23]
Sequence generation (selection bias)	Low:	Low:
	“Randomization was stratified by study center with a 1:1 ratio for PGx and TAU group, with the use of a random list generated by a computer”	“Randomization was stratified by center with a 1:1 ratio for intervention and control group, using a computer-generated random list”
Location concealment (selection bias)	Low:	Low:
	Randomization list created at an independent center	Randomization list created at an independent center
Blinding of participants and researchers (performance bias)	High:	High:
	Patients blinded	Patients blinded
	Treating clinician unblinded	Treating clinician unblinded
Blinding of outcome assessment (detection bias)	High:	High:
	CGI-S and HDRS-17 evaluated by the unblinded treating clinician	CGI-S and HDRS-17 evaluated by the unblinded treating clinician
Incomplete outcome data (attrition bias)	Low:	Low:
	Patients lost to follow-up were evenly distributed	Patients lost to follow-up were evenly distributed
Selective reporting (reporting bias)	Low:	Low:
	Prespecified outcomes were reported	Prespecified outcomes were reported
Other sources of bias	High:	High:
	Patients recruited by the treating clinician	Patients recruited by the treating clinician
	Non-industry sponsored	Industry sponsored

**Table 4:** Assessment of the risk of bias of the retrospective GENEPSI study.

Bias	GENEPSI Study [25]
Confounding (allocation bias)	Low:
	“Patient data were retrospectively pooled from three psychiatric clinics in Madrid (see author affiliations) that had been using the Neuropharmagen test”
	Balanced distribution regarding baseline severity (CGI-S), age, sex, substance abuse and concomitant diseases
	“Genetic testing could also result in increased placebo effect. In order to avoid these confounder effects, we sought to perform this retrospective study exclusively in patients who had been genotyped, rather than comparing patients who received pharmacogenetic testing to patients treated as usual.”
Selection of participants (Inception bias)	Low:
	Baseline visit established as the one in which the saliva sample from the patient was collected
	Follow-up visit established 12-weeks after the baseline visit
Misclassification of interventions (misclassification bias)	Moderate:
	Intervention status well defined, although determined retrospectively
Deviations from intended interventions (performance bias)	Low:
	Retrospective assignment of intervention. No differences between groups in the care provided
Missing data (attrition bias)	Low:
	Patients lost to follow-up were evenly distributed
Measurement of outcomes (detection bias)	Moderate:
	CGI-S recorded in the clinical history by the treating clinician prior to retrospective study protocol definition
Selective reporting (outcome reporting bias)	Low:
	Prespecified outcomes were reported

## Supplementary Materials

The following are available online at https://www.mdpi.com/1999-4923/11/9/453/s1, Table S1: List of genes and polymorphisms analyzed, Figure S1: Example of a Neuropharmagen^®^ pharmacogenomics interpretative report for one de-identified study subject, showing (**a**) the color-coding classification of drugs and (**b**) detailed information for one of the drugs.

## References

[1] WHO (2017). Depression and Other Common Mental Disorders.

[2] Steel Z., Marnane C., Iranpour C., Chey T., Jackson J.W., Patel V., Silove D. (2014). The global prevalence of common mental disorders: A systematic review and meta-analysis 1980–2013. Int. J. Epidemiol..

[3] König H., König H.-H., Konnopka A. (2019). The excess costs of depression: A systematic review and meta-analysis. Epidemiol. Psychiatr. Sci..

[4] Vos T., Abajobir A.A., Abate K.H., Abbafati C., Abbas K.M., Abd-Allah F., Abdulkader R.S., Abdulle A.M., Abebo T.A., Abera S.F. (2017). Global, regional, and national incidence, prevalence, and years lived with disability for 328 diseases and injuries for 195 countries, 1990–2016: A systematic analysis for the Global Burden of Disease Study 2016. Lancet.

[5] Rush A.J., Trivedi M.H., Wisniewski S.R., Nierenberg A.A., Stewart J.W., Warden D., Niederehe G., Thase M.E., Lavori P.W., Lebowitz B.D. (2006). Acute and Longer-Term Outcomes in Depressed Outpatients Requiring One or Several Treatment Steps: A STAR*D Report. Am. J. Psychiatry.

[6] Fournier J.C., DeRubeis R.J., Hollon S.D., Dimidjian S., Amsterdam J.D., Shelton R.C., Fawcett J. (2010). Antidepressant drug effects and depression severity: A patient-level meta-analysis. JAMA.

[7] Johnston K.M., Powell L.C., Anderson I.M., Szabo S., Cline S. (2019). The burden of treatment-resistant depression: A systematic review of the economic and quality of life literature. J. Affect. Disord..

[8] Sheehan D.V., Keene M.S., Eaddy M., Krulewicz S., Kraus J.E., Carpenter D.J. (2008). Differences in medication adherence and healthcare resource utilization patterns: Older versus newer antidepressant agents in patients with depression and/or anxiety disorders. CNS Drugs.

[9] Warden D., Rush A.J., Trivedi M.H., Fava M., Wisniewski S.R. (2007). The STAR*D Project results: A comprehensive review of findings. Curr. Psychiatry Rep..

[10] Kelly K., Posternak M., Alpert J.E. (2008). Toward achieving optimal response: Understanding and managing antidepressant side effects. Dialogues Clin. Neurosci..

[11] Swen J.J., Nijenhuis M., de Boer A., Grandia L., Maitland-van der Zee A.H., Mulder H., Rongen G.A., van Schaik R.H., Schalekamp T., Touw D.J. (2011). Pharmacogenetics: From bench to byte—An update of guidelines. Clin. Pharmacol. Ther..

[12] Leckband S.G., Kelsoe J.R., Dunnenberger H.M., George A.L., Tran E., Berger R., Muller D.J., Whirl-Carrillo M., Caudle K.E., Pirmohamed M. (2013). Clinical Pharmacogenetics Implementation Consortium Guidelines for HLA-B Genotype and Carbamazepine Dosing. Clin. Pharmacol. Ther..

[13] Caudle K.E., Rettie A.E., Whirl-Carrillo M., Smith L.H., Mintzer S., Lee M.T., Klein T.E., Callaghan J.T. (2014). Clinical Pharmacogenetics Implementation Consortium Guidelines for CYP2C9 and HLA-B Genotypes and Phenytoin Dosing. Clin. Pharmacol. Ther..

[14] Hicks J.K., Sangkuhl K., Swen J.J., Ellingrod V.L., Muller D.J., Shimoda K., Bishop J.R., Kharasch E.D., Skaar T.C., Gaedigk A. (2017). Clinical pharmacogenetics implementation consortium guideline (CPIC) for CYP2D6 and CYP2C19 genotypes and dosing of tricyclic antidepressants: 2016 update. Clin. Pharmacol. Ther..

[15] Hicks J.K., Bishop J.R., Sangkuhl K., Muller D.J., Ji Y., Leckband S.G., Leeder J.S., Graham R.L., Chiulli D.L., LLerena A. (2015). Clinical Pharmacogenetics Implementation Consortium (CPIC) guideline for CYP2D6 and CYP2C19 genotypes and dosing of selective serotonin reuptake inhibitors. Clin. Pharmacol. Ther..

[16] Caudle K.E., Keeling N.J., Klein T.E., Whirl-Carrillo M., Pratt V.M., Hoffman J.M. (2018). Standardization can accelerate the adoption of pharmacogenomics: Current status and the path forward. Pharmacogenomics.

[17] Bank P.C.D., Caudle K.E., Swen J.J., Gammal R.S., Whirl-Carrillo M., Klein T.E., Relling M.V., Guchelaar H.J. (2018). Comparison of the Guidelines of the Clinical Pharmacogenetics Implementation Consortium and the Dutch Pharmacogenetics Working Group. Clin. Pharmacol. Ther..

[18] Drozda K., Muller D.J., Bishop J.R. (2014). Pharmacogenomic testing for neuropsychiatric drugs: Current status of drug labeling, guidelines for using genetic information, and test options. Pharmacotherapy.

[19] Ehmann F., Caneva L., Prasad K., Paulmichl M., Maliepaard M., Llerena A., Ingelman-Sundberg M., Papaluca-Amati M. (2015). Pharmacogenomic information in drug labels: European Medicines Agency perspective. Pharmacogenom. J..

[20] Haddow J.E., Palomaki G.E., Khoury M.J., Little J., Burke W. (2004). ACCE: A Model Process for Evaluating Data on Emerging Genetic Tests. Human Genome Epidemiology: A Scientific Foundation for Using Genetic Information to Improve Health and Prevent Disease.

[21] Bousman C.A., Hopwood M. (2016). Commercial pharmacogenetic-based decision-support tools in psychiatry. Lancet Psychiatry.

[22] Wisniewski S.R., Rush A.J., Nierenberg A.A., Gaynes B.N., Warden D., Luther J.F., McGrath P.J., Lavori P.W., Thase M.E., Fava M. (2009). Can phase III trial results of antidepressant medications be generalized to clinical practice? A STAR*D report. Am. J. Psychiatry.

[23] Perez V., Salavert A., Espadaler J., Tuson M., Saiz-Ruiz J., Saez-Navarro C., Bobes J., Baca-Garcia E., Vieta E., Olivares J.M. (2017). Efficacy of prospective pharmacogenetic testing in the treatment of major depressive disorder: Results of a randomized, double-blind clinical trial. BMC Psychiatry.

[24] Han C., Wang S.-M., Bahk W.-M., Lee S.-J., Patkar A.A., Masand P.S., Mandelli L., Pae C.-U., Serretti A. (2018). A Pharmacogenomic-based Antidepressant Treatment for Patients with Major Depressive Disorder: Results from an 8-week, Randomized, Single-blinded Clinical Trial. Clin. Psychopharmacol. Neurosci..

[25] Espadaler J., Tuson M., Lopez-Ibor J.M., Lopez-Ibor F., Lopez-Ibor M.I. (2017). Pharmacogenetic testing for the guidance of psychiatric treatment: A multicenter retrospective analysis. CNS Spectr..

[26] Guy W. (1976). ECDEU Assessment Manual for Psychopharmacology: Revised.

[27] Hamilton M. (1960). A rating scale for depression. J. Neurol. Neurosurg. Psychiatry.

[28] Liberati A., Altman D.G., Tetzlaff J., Mulrow C., Gotzsche P.C., Ioannidis J.P.A., Clarke M., Devereaux P.J., Kleijnen J., Moher D. (2009). The PRISMA statement for reporting systematic reviews and meta-analyses of studies that evaluate healthcare interventions: Explanation and elaboration. BMJ.

[29] Porcelli S., Fabbri C., Serretti A. (2012). Meta-analysis of serotonin transporter gene promoter polymorphism (5-HTTLPR) association with antidepressant efficacy. Eur. Neuropsychopharmacol..

[30] Niitsu T., Fabbri C., Bentini F., Serretti A. (2013). Pharmacogenetics in major depression: A comprehensive meta-analysis. Prog. Neuro-Psychopharmacol. Biol. Psychiatry.

[31] Breitenstein B., Scheuer S., Bruckl T.M., Meyer J., Ising M., Uhr M., Holsboer F. (2016). Association of ABCB1 gene variants, plasma antidepressant concentration, and treatment response: Results from a randomized clinical study. J. Psychiatr. Res..

[32] Uhr M., Tontsch A., Namendorf C., Ripke S., Lucae S., Ising M., Dose T., Ebinger M., Rosenhagen M., Kohli M. (2008). Polymorphisms in the drug transporter gene ABCB1 predict antidepressant treatment response in depression. Neuron.

[33] van der Weide K., van der Weide J. (2014). The influence of the CYP3A4*22 polymorphism on serum concentration of quetiapine in psychiatric patients. J. Clin. Psychopharmacol..

[34] Mas S., Gasso P., Ritter M.A., Malagelada C., Bernardo M., Lafuente A. (2015). Pharmacogenetic predictor of extrapyramidal symptoms induced by antipsychotics: Multilocus interaction in the mTOR pathway. Eur. Neuropsychopharmacol..

[35] Hedges L. (1981). V Distribution Theory for Glass’s Estimator of Effect size and Related Estimators. J. Educ. Behav. Stat..

[36] Schwarzer G., Carpenter J.R., Rücker G. (2015). Meta-Analysis with R.

[37] Pishva E., Drukker M., Viechtbauer W., Decoster J., Collip D., van Winkel R., Wichers M., Jacobs N., Thiery E., Derom C. (2014). Epigenetic genes and emotional reactivity to daily life events: A multi-step gene-environment interaction study. PLoS ONE.

[38] RStudio Team (2015). RStudio: Integrated Development for R.

[39] R Core Team (2015). R: A Language and Environment for Statistical Computing.

[40] Higgins J.P.T., Thompson S.G., Deeks J.J., Altman D.G. (2003). Measuring inconsistency in meta-analyses. BMJ.

[41] Hasselblad V., Hedges L.V. (1995). Meta-Analysis of Screening and Diagnostic Tests. Psychol. Bull..

[42] Chinn S. (2000). A simple method for converting an odds ratio to effect size for use in meta-analysis. Stat. Med..

[43] Higgins J.P.T., Altman D.G., Gøtzsche P.C., Jüni P., Moher D., Oxman A.D., Savovic J., Schulz K.F., Weeks L., Sterne J.A.C. (2011). The Cochrane Collaboration’s tool for assessing risk of bias in randomised trials. BMJ.

[44] Sterne J.A., Hernán M.A., Reeves B.C., Savović J., Berkman N.D., Viswanathan M., Henry D., Altman D.G., Ansari M.T., Boutron I. (2016). ROBINS-I: A tool for assessing risk of bias in non-randomised studies of interventions. BMJ.

[45] Gibertini M., Nations K.R., Whitaker J.A. (2012). Obtained effect size as a function of sample size in approved antidepressants. Int. Clin. Psychopharmacol..

[46] Cipriani A., Furukawa T.A., Salanti G., Chaimani A., Atkinson L.Z., Ogawa Y., Leucht S., Ruhe H.G., Turner E.H., Higgins J.P.T. (2018). Comparative efficacy and acceptability of 21 antidepressant drugs for the acute treatment of adults with major depressive disorder: A systematic review and network meta-analysis. Lancet.

[47] Zimmerman M., Mattia J.I., Posternak M.A. (2002). Are Subjects in Pharmacological Treatment Trials of Depression Representative of Patients in Routine Clinical Practice?. Am. J. Psychiatry.

[48] Zetin M., Hoepner C.T. (2007). Relevance of Exclusion Criteria in Antidepressant Clinical Trials. J. Clin. Psychopharmacol..

[49] Blanco C., Olfson M., Goodwin R.D., Ogburn E., Liebowitz M.R., Nunes E.V., Hasin D.S. (2008). Generalizability of clinical trial results for major depression to community samples: Results from the National Epidemiologic Survey on Alcohol and Related Conditions. J. Clin. Psychiatry.

[50] Kessler R.C., Berglund P., Demler O., Jin R., Koretz D., Merikangas K.R., Rush A.J., Walters E.E., Wang P.S. (2003). National Comorbidity Survey Replication the Epidemiology of Major Depressive Disorder. JAMA.

[51] Hasin D.S., Goodwin R.D., Stinson F.S., Grant B.F. (2005). Epidemiology of Major Depressive Disorder. Arch. Gen. Psychiatry.

[52] Zimmerman M., Chelminski I., McDermut W. (2002). Major depressive disorder and axis I diagnostic comorbidity. J. Clin. Psychiatry.

[53] Melartin T.K., Rytsälä H.J., Leskelä U.S., Lestelä-Mielonen P.S., Sokero T.P., Isometsä E.T. (2002). Current comorbidity of psychiatric disorders among DSM-IV major depressive disorder patients in psychiatric care in the Vantaa Depression Study. J. Clin. Psychiatry.

[54] Rush A.J. (2007). STAR*D: What Have We Learned?. Am. J. Psychiatry.

[55] Romera I., Pérez V., Menchón J.M., Polavieja P., Gilaberte I. (2011). Optimal cutoff point of the Hamilton Rating Scale for Depression according to normal levels of social and occupational functioning. Psychiatry Res..

[56] Cameron I.M., Reid I.C., MacGillivray S.A. (2014). Efficacy and tolerability of antidepressants for sub-threshold depression and for mild major depressive disorder. J. Affect. Disord..

[57] Singh A.B. (2015). Improved Antidepressant Remission in Major Depression via a Pharmacokinetic Pathway Polygene Pharmacogenetic Report. Clin. Psychopharmacol. Neurosci..

[58] Bradley P., Shiekh M., Mehra V., Vrbicky K., Layle S., Olson M.C., Maciel A., Cullors A., Garces J.A., Lukowiak A.A. (2018). Improved efficacy with targeted pharmacogenetic-guided treatment of patients with depression and anxiety: A randomized clinical trial demonstrating clinical utility. J. Psychiatr. Res..

[59] Rush A.J., Fava M., Wisniewski S.R., Lavori P.W., Trivedi M.H., Sackeim H.A., Thase M.E., Nierenberg A.A., Quitkin F.M., Kashner T.M. (2004). Sequenced treatment alternatives to relieve depression (STAR*D): Rationale and design. Control. Clin. Trials.

[60] Menchon J.M., Espadaler J., Tuson M., Saiz-Ruiz J., Bobes J., Vieta E., Alvarez E., Perez V. (2019). Patient characteristics driving clinical utility in psychiatric pharmacogenetics: A reanalysis from the AB-GEN multicentric trial. J. Neural Transm..

[61] Bousman C.A., Arandjelovic K., Mancuso S.G., Eyre H.A., Dunlop B.W. (2019). Pharmacogenetic tests and depressive symptom remission: A meta-analysis of randomized controlled trials. Pharmacogenomics.

[62] Rosenblat J.D., Lee Y., McIntyre R.S. (2018). The effect of pharmacogenomic testing on response and remission rates in the acute treatment of major depressive disorder: A meta-analysis. J. Affect. Disord..

[63] Bousman C.A., Zierhut H., Müller D.J. (2019). Navigating the Labyrinth of Pharmacogenetic Testing: A Guide to Test Selection. Clin. Pharmacol. Ther..

[64] Bousman C.A., Dunlop B.W. (2018). Genotype, phenotype, and medication recommendation agreement among commercial pharmacogenetic-based decision support tools. Pharmacogenom. J..

[65] Zeier Z., Carpenter L.L., Kalin N.H., Rodriguez C.I., McDonald W.M., Widge A.S., Nemeroff C.B. (2018). Clinical Implementation of Pharmacogenetic Decision Support Tools for Antidepressant Drug Prescribing. Am. J. Psychiatry.

