# Supplementary material for: Effectiveness of a Pharmacogenetic Tool at Improving Treatment Efficacy in Major Depressive Disorder: A Meta-Analysis of Three Clinical Studies
Source: Pharmaceutics. 2019 Sep 2;11(9):453. doi: 10.3390/pharmaceutics11090453 (PMC6781283; doi:10.3390/pharmaceutics11090453)
Supplement: Supplementary file 1 [file pharmaceutics-11-00453-s001.pdf]

pharmaceutics

# Supplementary Materials: Effectiveness of a Pharmacogenetic Tool at Improving Treatment Efficacy in Major Depressive Disorder: A Meta-Analysis of Three Clinical Studies

Silvia Vilches, Miquel Tuson, Eduard Vieta, Enric Álvarez and Jordi Espadaler

**Table S1.** List of genes and polymorphisms analyzed.

| Gene symbol | Gene name | Polymorphisms |
|---|---|---|
| *ABCB1* | ATP binding cassette subfamily B member 1 | rs2235048, rs11983225 |
| *AKT1* | V-akt murine thymoma viral oncogene homolog 1 | rs1130214 |
| *BDNF* | Brain-derived neurotrophic factor | rs6265 |
| *CACNG2* | Calcium channel, voltage-dependent, gamma subunit 2 | rs2284017 |
| *CES1* | Carboxylesterase 1 | rs71647871 |
| *COMT* | Catechol-O-methyltransferase | rs4680 |
| *CRHR1* | Corticotropin releasing hormone receptor 1 | rs4792888 |
| *CYP1A2* | Cytochrome P450 family 1 subfamily A member 2 | *1, *1F |
| *CYP2B6* | Cytochrome P450 family 2 subfamily B member 6 | *1, *6 |
| *CYP2C19* | Cytochrome P450 family 2 subfamily C member 19 | *1, *2, *3, *5, *7, *8, *17, *27 |
| *CYP2C9* | Cytochrome P450 family 2 subfamily C member 9 | *1, *2, *3, *6, *8, *27 |
| *CYP2D6* | Cytochrome P450 family 2 subfamily D member 6 | *1, *2, *3, *4, *5, *6, *7, *8, *9, *10, *11, *12, *14, *15, *17, *19, *20, *29, *30, *35, *40, *41, *69, *1xN, *2xN, *35X2 |
| *CYP3A4* | Cytochrome P450 family 3 subfamily A member 4 | *1, *22 |
| *DDIT4* | DNA damage inducible transcript 4 | rs1053639 |
| *DRD3* | Dopamine receptor D3 | rs963468 |
| *EPHX1* | Epoxide hydrolase 1, microsomal (xenobiotic) | rs1051740 |
| *FCHSD1* | FCH and double SH3 domains 1 | rs456998 |
| *GRIK2* | glutamate receptor, ionotropic, kainate 2 | rs2518224 |
| *GRIK4* | glutamate receptor, ionotropic kainate 4 | rs1954787 |
| *HLA-A* | Major histocompatibility complex, class I, A | rs1061235 |
| *HTR1A* | 5-HT (serotonin) receptor 1A, G protein-coupled | rs10042486 |
| *HTR2A* | 5-HT (serotonin) receptor 2A, G protein-coupled | rs6311, rs6314, rs9316233 |
| *HTR2C* | 5-HT (serotonin) receptor 2C, G protein-coupled | rs1414334 |
| *LPHN3* | Latrophilin 3 | rs6551665 |
| *NEFM* | Neurofilament, medium polypeptide | rs1379357, rs1457266 |
| *OPRM1* | Opioid receptor, mu 1 | rs1799971 |
| *RGS4* | Regulator of G-protein signaling 4 | rs2661319 |
| *RPTOR* | Regulatory associated protein of MTOR, complex 1 | rs7211818 |
| *SLC6A4* | Solute carrier family 6 (neurotransmitter transporter), member 4 | 5-HTTLPR |
| *UGT2B15* | UDP glucuronosyltransferase 2 family, polypeptide B15 | rs1902023 |

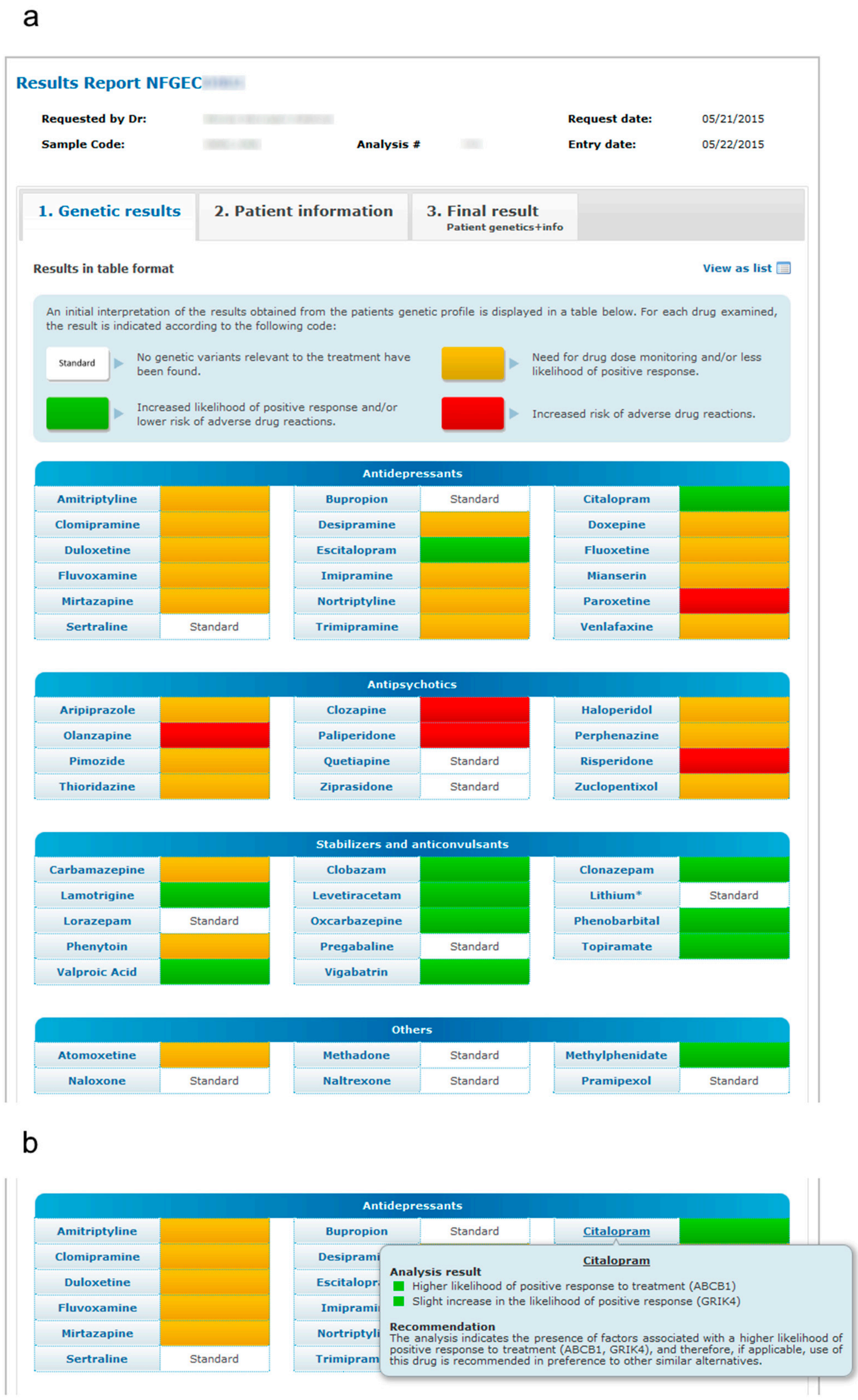


**Figure S1.** Example of a Neuropharmagen® pharmacogenomics interpretative report for one de-identified study subject, showing (**a**) the color-coding classification of drugs and (**b**) detailed information for one of the drugs.
